# Ocular and Systemic Effects of Antioxidative Supplement Use in Young and Healthy Adults: Real-World Cross-Sectional Data

**DOI:** 10.3390/antiox9060487

**Published:** 2020-06-03

**Authors:** Sakiko Minami, Norihiro Nagai, Misa Suzuki, Atsuro Uchida, Hajime Shinoda, Kazuo Tsubota, Yoko Ozawa

**Affiliations:** 1 Department of Ophthalmology, Keio University School of Medicine, 35 Shinanomachi, Shinjuku-ku, Tokyo 160-8582, Japan; saki.love5@icloud.com (S.M.); nagai@a5.keio.jp (N.N.); misa.suzuki@suzukiganka.com (M.S.); uchidats@gmail.com (A.U.); shinoha@mac.com (H.S.); tsubota@z3.keio.jp (K.T.); 2 Laboratory of Retinal Cell Biology, Keio University School of Medicine, 35 Shinanomachi, Shinjuku-ku, Tokyo 160-8582, Japan; 3 Department of Ophthalmology, St. Luke’s International Hospital, 9-1 Akashi-cho, Chuo-ku, Tokyo 104-8560, Japan; 4 St. Luke’s International University, 9-1 Akashi-cho, Chuo-ku, Tokyo 104-8560, Japan

**Keywords:** antioxidative supplement, visual acuity, hs-CRP, cholesterol, HbA1c

## Abstract

Randomized controlled studies have shown that antioxidative supplements are effective in suppressing the progression of age-related macular degeneration and visual display terminal syndrome. However, effects of their general use in the real-world and by young and healthy individuals have not been well documented. We analyzed 27 participants who were under 35 years of age and had no diagnosed diseases. Mean functional visual acuity (FVA) score and visual maintenance ratio, which represent quick recognition of a target, both measured using FVA system, were better (both *p* < 0.01) in subjects who had had regular antioxidative supplement intake for more than 2 months (11 participants) compared with those who had not. Systemic data, i.e., total cholesterol, hemoglobin A1c (HbA1c), and high-sensitivity C-reactive protein (hs-CRP) levels, which correspond to chronic low-grade inflammation, were lower (all *p* < 0.05) in the former. Overall, hs-CRP levels had a correlation with total cholesterol (*p* < 0.05) and a trend of correlation with HbA1c (*p* = 0.054) levels. Thus, current real-world data showed that young, healthy participants who had a regular intake of antioxidative supplements had better visual acuity and systemic levels of metabolic and low-grade inflammation markers. This study will help promote future research into the effects of general antioxidative supplement use.

## 1. Introduction

Recent increased interest in health care has led to the general public paying more attention to antioxidative supplements for the maintenance of their health, e.g., for eye and systemic conditions. In fact, there is a reported correlation between serum levels of antioxidants, such as vitamins and carotenoids, and the prevalence of age-related macular degeneration (AMD) [1], and several large-scale randomized controlled trials (RCTs) have demonstrated the effects of antioxidative supplements in preventing AMD progression [2,3]. In addition, several small-scale intervention studies have shown their effectiveness in mitigating discomfort and/or eye fatigue in visual display terminal syndrome [4,5,6]. Regarding systemic diseases, a meta-analysis of clinical studies has revealed the effects of spirulina, which contains various antioxidative components, on the improvement of dyslipidemia [7]. However, effects of antioxidative supplements in a general and in the real-world context remain unclear. In contrast to in RCTs, the choice of supplements and intake schedules are in the hands of individuals in the real-world. In particular, the effects of supplements in young and healthy people are not well-documented, although the number of individuals in the general public taking supplements is growing [8].

Fundamental eye health is often evaluated based on visual acuity. This is usually measured using the Snellen or Landolt C charts, and respective scores over 20/20 or 1.0 on the decimal scale are required for having healthy eyes with no eye disease. Nonetheless, even when visual acuity scores are the same, there are sometimes complaints of visual impairment with the best correction of the refractive errors. Thus, variations in visual ability exist. The variations in visual function among healthy individuals were previously shown using an objective method and an electrophysiological test [9]. However, the Snellen or Landolt C charts do not detect these variations, thus, they are not suitable to detect variations in eye health levels of individuals who do or do not take supplements. We have previously reported that the functional visual acuity (FVA) system [10] can detect slight changes in visual ability, even if it is preserved completely when based on the Snellen or Landolt C chart [11,12]. In contrast to the Snellen or Landolt C chart methods, where there is no strict restriction on the time taken to observe the optotypes for measurement, participants have to continuously respond to optotypes in the FVA system, and the optotypes are automatically changed every 2 s during the 60-s test period. Thus, people who take a longer time to recognize the optotypes have a lower visual ability based on the FVA system. This is also different from contrast visual acuity, which is measured using low-contrast optotypes and can detect slight changes in those who had good visual acuity, such as a 20/20 score [11]; when measuring contrast visual acuity, the individual stares at the optotypes for some time, similar to the Snellen or Landolt C chart methods. Morphological changes of the neural retina, where photoreceptor and subsequent neural networks are involved, can be detected by optical coherence tomography (OCT), and its three-dimensional recordings enable us to evaluate the neural volume. Retinal volume also varies among individuals who have no diagnosed eye diseases [9,13]. 

Due to the recent increase in the number of patients with metabolic syndrome [14,15], unaffected individuals who have not been diagnosed with diabetes or dyslipidemia have increasingly begun to pay attention to their blood glucose and/or cholesterol levels and worry about their risks of future disease. This phenomenon has also become more evident in Japan with the Westernization of the daily diet [16]. The results that vitamin D [17] and magnesium [18] reduced blood glucose levels in patients with diabetes were also reported on websites accessible to the general public, for example, on the websites of suppliers of the commercially available supplements. However, systemic data on the effects of commercially available supplements used in the real-world are seldom provided.

High-sensitivity C-reactive protein (hs-CRP; [19,20]) as well as oxidized low-density lipoprotein (MDL-LDL; [21,22,23]) are biomarkers for the risk of age-related and metabolic disorders such as coronary heart disease and cardiovascular events. High levels of hs-CRP also increase the risk of type 2 diabetes [24], non-alcoholic fatty liver disease [25], and AMD [26,27]. While CRP is synthesized in the liver and its serum levels increase substantially in response to acute inflammation, hs-CRP can indicate low-grade inflammation [7]. It has been reported that vitamin C can lower hs-CRP levels in participants with high hs-CRP levels, those younger than 60 years old, or those that used intravenous administration of vitamin C [28]. Meanwhile, commercially available supplements are mixtures of several antioxidants, and their effects on hs-CRP levels have not been investigated.

In the current study, we measured FVA scores and visual maintenance ratios (VMRs) using the FVA system and systemic data using blood samples to estimate the effects of commercially available general supplements on young and healthy adults. 

## 2. Materials and Methods 

This study was conducted according to the guidelines of the Declaration of Helsinki. All procedures involving human subjects were approved by the Ethics Committee of Keio University School of Medicine (Approval No. 20100295). Written informed consent was obtained from all subjects.

### 2.1. Subjects

This study was performed at the Medical Retina Division, Department of Ophthalmology, Keio University School of Medicine from August to September 2019. Healthy Japanese volunteers without any ocular diseases were considered eligible. Participants older than 35 years of age were excluded. The participants were not selected if they had a habit of antioxidant intake. The final sample comprised 27 people who agreed to sign the informed consent form. Histories of ocular and systemic diseases and general habits, i.e., smoking and regular intake of micronutrient supplements, were reviewed. 

### 2.2. Ophthalmologic Examinations

All included subjects underwent the best-corrected visual acuity (BCVA) measurement using a Landolt C chart, refraction test, intraocular pressure (IOP) measurement, fundus examination, and OCT recordings. The BCVA was converted to express on the log scale using the logMAR method. The FVA was measured using an FVA measurement system (Nidek, Tokyo, Japan) with best correction as described elsewhere [29]. Briefly, Landolt optotypes were presented on the monitor, and their size was changed every 2 s for 60 s depending on the correctness of the responses—if the response was correct, smaller optotypes were presented, and if it was incorrect, larger optotypes were presented; when there was no response within 2 s, the next optotype was automatically enlarged. FVA scores represent mean FVA during 60 s. To compare the changes in VA over time, the VMR was determined as follows: VMR = (lowest logMAR VA score − FVA at 60 s)/ (lowest logMAR VA score − baseline VA). The contrast VA was tested with best correction using CSV-1000 LanC charts shown in low contrast that were 6% or 12% of the usual optotypes (VectorVision, Inc., Greenville, OH, USA), and the values were recorded in log scale [11,12]. This test was performed in eyes with undilated pupils at a testing distance of 2.5 m under best spectacle correction. Background illumination of the translucent chart was provided by a fluorescent source in the instrument and was automatically calibrated to 85 cd/m^2^. OCT was performed using a Heidelberg Spectralis OCT system (Heidelberg Engineering GmbH, Dossenheim, Germany), and the retinal volumes were measured using three-dimensional recordings. All ocular examinations were performed monocularly in their left eyes.

### 2.3. Blood Sample Test

Blood samples were obtained, and measurements for clinical biochemistry, hematology, and hs-CRP levels were performed at SRL Inc. (Tokyo, Japan). HDL, LDL, and MDA-LDL were measured by enzyme-linked immunosorbent assay, TG was by glycerol-3-phosphate -peroxidase chromogenic method, T-Cho was by cholesterol oxidase-peroxidase-voltammetry system, hemoglobin A1c (HbA1c) and hemoglobin (Hb) were by enzymatic assay, AST(GOT), ALT(GPT) and γ-GTP were by JSCC reference method, WBC, RBC and Plt were by flowcytometry, Ht, MCV, MCH and MCHC were by calculation method, and hs-CRP was by latex coagulating nephelometry.

### 2.4. Ingestion Frequency Investigation

The dietary intake of each nutrient and total caloric intake were determined using a self-administered questionnaire on ingestion frequency; this questionnaire has been validated for dietary investigations in the Japanese (Education Software Co.,Ltd., Tokyo, Japan [30,31]).

### 2.5. Statistical Analyses

All results are expressed as the mean ± standard error (SE). Commercial statistical software (SPSS; ver. 25, SPSS Inc., IBM Corp, Armonk, NY, USA) was used for the analyses. *t*-test, Fisher’s exact test, Mann–Whitney U test, and Pearson product-moment correlation coefficient were performed. Differences were considered statistically significant at *p* < 0.05.

## 3. Results

Mean age of the 27 participants was 26.6 ± 0.8 (range, 21 to 35) years, and 9 males (33%) were included (Table 1). BCVA of all the participants was −0.08 in LogMAR, and mean IOP was 13.5 ± 0.4 mmHg. Five participants (19%) had a smoking habit, 21 (78%) had a drinking habit, 19 (70%) had a snacking habit, and 10 (37%) had an exercise habit. Eleven (44%) participants had ingested commercially available antioxidative supplements (Table 2), such as bilberry extracts and/or multivitamins, daily and regularly for more than 2 months prior to the current study (Table 1).

Next, we divided the participants into 2 groups according to the habit of taking antioxidative supplements for more than 2 months (Table 3). There were no differences in age, sex, BCVA measured using Landolt C chart, IOP, and the other abovementioned habits, other than taking supplements, between the two groups. However, there were significant differences in FVA score and VMR; the respective FVA scores of non-supplement intake group and supplement intake group were 0.13 ± 0.03 and −0.01 ± 0.02 (*p* = 0.004), and VMR scores were 0.87 ± 0.02 and 0.96 ± 0.01 (*p* = 0.007). Thus, the values were significantly better in the supplement intake group (Table 3). Contrast VA and OCT data were not significantly different (Table 3).

We also analyzed the systemic data. We found significant differences between the two groups in terms of the T-Chol (non-supplement intake group vs. supplement intake group: 191.50 ± 6.30 vs. 168.64 ± 6.28 (mg/dL), *p* = 0.020), HbA1c (non-supplement intake group vs. supplement intake group: 5.33 ± 0.09 vs. 5.13 ± 0.04 (%), *p* = 0.048), and more evidently, hs-CRP (non-supplement intake group vs. supplement intake group: 1197.38 ± 436.07 vs. 134.44 ± 40.53 (ng/mL), *p* = 0.034) levels; all the values were significantly lower in the supplement intake group (Table 4).

Overall, hs-CRP level was negatively correlated with that of HDL (*r* = −0.389, *p* = 0.045), and positively correlated with that of LDL (*r* = 0.625, *p* < 0.001), LDL/ HDL ratio (*r* = 0.748, *p* < 0.001), MDA-LDL (*r* = 0.468, *p* = 0.014), T-Chol (*r* = 0.511, *p* = 0.006), ALT (*r* = 0.412, *p* = 0.033), and γGTP (*r* = 0.585, *p* = 0.0015), and was related to lipid and liver metabolism (Table 5). Moreover, there was a trend of correlation between hs-CRP and HbA1c levels (*r* = 0.375, *p* = 0.054).

Results of the ingestion frequency investigation showed no differences in the groups regarding total caloric, carbohydrate, lipid, and protein intake from the daily meals, while vitamin D intake was less in the supplement intake group (*p* = 0.047) (Table 6). However, the levels of daily vitamin D intake were comparable between the two groups when the amount of vitamin D contained in the supplements was added to that contained in daily meals in the supplement intake group (6.15 ± 1.38 μg/day, *p* = 0.789, data not shown).

## 4. Discussion

Visual function measured using the FVA system, i.e., FVA score and VMR, was superior, and systemic T-Chol, HbA1c, and hs-CRP levels were lower in the young healthy adults who had regular intake of antioxidative supplements for more than 2 months. In particular, hs-CRP values, while they were all within the normal range, were one digit lower in the supplement intake group. Overall, hs-CRP level had correlations with lipid and liver metabolism, and a trend of correlation with HbA1c.

The effectiveness of antioxidative supplements in preventing the progression of AMD [2,3], a blinding disease, have been previously reported; however, these effects were assessed and observed only in patients who already had signs of AMD, and were thus defined as high-risk patients. In contrast, the effects in the eyes of general participants with no serious diseases were reported regarding fatigue or accommodation disorders such as visual display terminal syndrome [6,32,33], dry eye disorders [34], and asthenopia [35]. Therefore, the effects were generally thought to be on mitigating the uncomfortableness rather than affecting visual acuity. The current study was a cross-sectional observational study, and the supplements used varied among individuals. However, interestingly, the FVA score and VMR were clearly better in the participants who were habitually taking the supplements. The FVA score reflects the ability of quick recognition of optotypes, because the participants have to respond within 2 s. Thus, this ability was superior in those who took antioxidative supplements regularly compared with those who did not. The VMR, the integral of the FVA score during 60 s, was also superior in the group that took antioxidative supplements. Therefore, supplement intake may have a positive effect on the rapidness of recognition and its persistence.

Reduction in FVA score and VMR can be observed in patients with moderate cataract [36] or after cataract which is the opacity of the lens capsule developed after cataract extraction [37], slight macular disorders due to mild AMD [12] and epiretinal membrane [11], severe dry eyes due to Sjogren syndrome [10] and Stevens Johnson syndrome [38], and post LASIK [39]. However, in the current study, there were no participants with such diseases. 

One objective method of measuring visual function, spatial-sweep steady-state pattern electroretinography, has revealed the variations of visual function among healthy adults with no diagnosed eye diseases [9]. In the same study, the visual function was found to be correlated with macular volume measured using OCT images; thus, the visual function reflected the variations of neural retinal morphology. Theoretically, better FVA among the healthy adults could also be related to retinal morphology, although there was no difference in the OCT measurements between participants with or without regular intake of supplements in the current study.

Alternatively, FVA score could be changed by using eye drop medication in patients with mild ocular surface disorders due to short tear break-up time even without corneal epithelial signs [40,41]. Therefore, supplements might have improved ocular surface condition, leading to better FVA scores. A previous report has shown that oral vitamin D supplementation may affect the ocular surface [42], although total vitamin D intake from daily meals and supplements in the supplement intake group was not more than that in the non-supplement intake group. Further study to assess the mechanism underlying changes in FVA scores and VMR due to antioxidative supplementation would be of value as a future project.

Participants who had been taking supplements regularly had better T-Chol, HbA1c, and hs-CRP levels. T-Chol and HbA1c could be affected by snacking and/or exercise habits; however, there were no differences in these habits between the groups. Moreover, there were no particular differences in the food intake as determined using the questionnaire.

Interestingly, hs-CRP levels were found to be correlated with those of T-Chol in the current study. A previous report showed that hs-CRP levels correlated positively with cardiometabolic risk factors, such as the T-Chol-to-HDL ratio, in Turkish children and adolescents [43]. Another study [44] has also discussed that dyslipidemia in childhood can trigger low-grade inflammation even in the absence of obesity. In contrast, inflammation can cause dyslipidemia by affecting ATP binding cassette A1-dependent cholesterol efflux [45]. Thus, inflammation and dyslipidemia can interact mutually. A correlation between hs-CRP and HbA1c levels has been reported for diabetic patients [46], and low grade inflammation and HbA1c could be related biologically. Further analyses to assess the relationships between antioxidative supplements and systemic data in young and healthy participants would be of value to know if they could facilitate avoiding future risks involving aging and metabolic diseases.

There was an obvious difference in the hs-CRP levels between the with and without regular intake of antioxidative supplements groups. High levels of hs-CRP are reported to be found in patients with diabetic retinopathy, AMD [47], and AMD high-risk variants of ARMS2/HTRA1 SNPs [48]. The values were all within the normal range; however, because the participants were all young, those who had relatively higher hs-CRP levels may be prone to abnormally high levels of hs-CRP when older. Future studies to investigate whether hs-CRP levels in young adults are related to the future development of eye diseases with age would be of interest. Animal experiments have shown that antioxidant intake reduces inflammatory signals in vivo [49,50,51,52,53,54], suggesting that regular antioxidative supplement intake could have reduced hs-CRP levels. Low-grade inflammation represented by higher levels of hs-CRP, while within the normal range, could have caused minimal neural disorganizations in the retina and/or ocular surface disorders in the participants who had no regular intake of antioxidative supplements, and thus, reduced FVA scores and VMR in the current study; nevertheless, further studies are required to clarify these aspects. 

Intake of each nutrient from food was not different between the two groups, except for that of vitamin D; its intake from daily meals was less in the supplement intake group. Dietary vitamin D sources include fungi, fish, meats, and eggs [55]. Due to recent changes in the Japanese diet, the intake of vegetables and fish has decreased and that of meats and oils has increased [16]. Thus, it is likely that all participants included in this study were in the habit of eating more meats and eggs than vegetables and fish. However, the supplement intake group may have consumed a lesser amount of eggs and/or meats to reduce fat intake from them, considering their health; the supplement intake group is also more likely to have higher levels of health awareness. Relatively lower intake of protein and fat was observed in the supplement intake group, which might support this idea. 

The limitations of the current study were the relatively small sample size, variations in the antioxidative supplements taken by the participants, and that supplement intake data were based on self-reports. The sunlight exposure habit, which is important for the in vivo conversion of vitamin D [56,57], was not assessed; furthermore, the levels of converted and circulating vitamin D, in the form of 25(OH)D, were not analyzed in the current study, although all participants were office workers and are not expected to be exposed to sunlight frequently. Intake of vitamin D may decrease the risk of cardiovascular diseases [58], thus may be an interesting future topic for general people. Further studies with more participants and more detailed serological measurements or interventional studies in participants with and without past histories of antioxidative supplement intake are warranted.

## 5. Conclusions

In the current study, young and healthy participants (below 35 years of age) with a regular intake of antioxidative supplements for more than 2 months had better visual function, as recorded using the FVA system, and were healthier systemically, as represented by lower T-Chol, HbA1c, and hs-CRP levels. The results may help propose a prospective intervention study with antioxidative supplements in young and healthy adults. Moreover, it will help determine the study protocol; it is worth measuring visual function using the FVA system, and not only conventional charts, as well as systemic data with respect to lipid and glucose metabolism, and more importantly, hs-CRP. The relationship between antioxidative supplements and hs-CRP levels would be an interesting topic for a basic research study. The current study will be informative for future clinical studies and will motivate basic studies on the effects of general antioxidative supplement use.

## Figures and Tables

**Table 1:** Characteristics of the participants.

Factors	*n* = 27
Age	26.6 ± 0.8
Sex (male %)	9 (33)
BCVA (LogMAR)	−0.08 ± 0.00
FVA score	0.08 ± 0.03
VMR	0.90 ± 0.02
Contrast VA 6%	0.28 ± 0.03
Contrast VA 12%	0.13 ± 0.03
Spherical Equivalent (Diopters)	−2.95 ± 0.65
Axial Length (mm)	24.91 ± 0.28
IOP (mmHg)	13.5 ± 0.4
Smoking habit (%)	5 (19)
Drinking habit (%)	21 (78)
Snacking habit (%)	19 (70)
Exercise habit (%)	10 (37)
Antioxidative supplement intake (%)	11 (41)
Duration of antioxidative supplement intake (Months) (range)	4.44 ± 1.89 (0–36)

Data are shown in mean ± standard error. BCVA, best-corrected visual acuity; VA, visual acuity; FVA, functional visual acuity; VMR, visual maintenance ratio; IOP, intraocular pressure.

**Table 2:** Main contents of the antioxidative supplements taken by individuals.

Participants	Contents
1	Resveratrol (Santa Berry extract) 4 mg
2	Anthocyanin (Bilberry extract) 160 mg, Multivitamin, Zinc 7 mg, Vitamin D 10.5 μg
3	Bifidobacteria, Malic acid
4	Anthocyanin (Bilberry extract) 160 mg, Bifidobacteria, Multivitamin
5	Anthocyanin (Bilberry extract) 160 mg
6	Anthocyanin (Bilberry extract) 160 mg
7	Vitamin C 2000 mg
8	Anthocyanin (Bilberry extract) 160 mg, Lutein 10 mg
9	Anthocyanin (Bilberry extract) 160 mg, Multivitamin, Zinc 3.5 mg
10	Anthocyanin (Bilberry extract) 160 mg, Multivitamin, Rose oil
11	Bifidobacteria, Theanine 200 mg

Multivitamin contains; vitamin C (260 mg), niacin (11 mg), vitamin E (8.0 mg), vitamin B2 (1.1 mg), vitaminB1 (1.0 mg), vitamin B6 (1.0 mg), vitamin D (5.0 μg), and vitamin B12 (2.0 μg).

**Table 3:** Ocular data of the participants with or without antioxidative supplement intake.

Factors	Non-Supplement Intake Group (*n* = 16)	Supplement Intake Group (*n* = 11)	*p*
Age	26.69 ± 0.93	32.57 ± 3.62	0.837
Sex (male, eyes %) ^†^	4 (25)	5 (46)	0.411
BCVA ^§^	−0.08 ± 0.00	−0.08 ± 0.00	1.000
FVA score	0.13 ± 0.03	−0.01 ± 0.02	0.004 **
VMR	0.87 ± 0.02	0.96 ± 0.01	0.007 **
Contrast VA 6%	0.28 ± 0.04	0.27 ± 0.04	0.834
Contrast VA 12%	0.12 ± 0.04	0.13 ± 0.04	0.863
Spherical Equivalent (D)	−3.26 ± 0.98	−2.51 ± 0.65	0.585
Axial length (mm)	25.06 ± 0.36	24.69 ± 0.42	0.529
IOP	13.85 ± 0.63	13.53 ± 0.70	0.287
OCT data			
MV (IRL)	3.99 ± 0.07	3.95 ± 0.07	0.691
MV (ORL)	4.38 ± 0.04	4.42 ± 0.05	0.527
MV (RPE)	0.38 ± 0.00	0.38 ± 0.04	0.863
Smoking habit (%) ^†^	1 (6)	4 (36)	0.125
Drinking habit (%) ^†^	11 (69)	10 (91)	0.350
Snacking habit (%) ^†^	10 (63)	9 (82)	0.405
Exercise habit (%) ^†^	6 (38)	4 (36)	1.000
Duration of antioxidative supplement intake (Months) (range)	0.19 ± 0.10 (0–1)	10.64 ± 4.03 (2–36)	0.004 **

Data are shown in mean ± standard error. ^†^ Fisher’s exact test; ^§^ Mann–Whitney U test; the others, *t*-test. BCVA, best-corrected visual acuity; VA, visual acuity; FVA, functional visual acuity; VMR, visual maintenance ratio; IOP, intraocular pressure, MV, macular volume; IRL, intraretinal layer; ORL. Outer retinal layer; RPE, retinal pigment epithelium. ** *p* <0.01.

**Table 4:** Systemic data of the participants with or without antioxidative supplement intake.

Factors	Non-Supplement Intake Group (*n* = 16)	Supplement Intake Group (*n* = 11)	*p*
HDL (mg/dL)	61.31 ± 4.77	62.27 ± 4.65	0.891
LDL (mg/dL)	111.94 ± 8.15	93.18 ± 5.22	0.065
LDL/ HDL ratio	2.02 ± 0.22	1.59 ± 0.15	0.159
MDA-LDL (U/L)	91.75 ± 7.66	79.18 ± 7.87	0.278
TG (mg/dL)	109.50 ± 20.17	90.36 ± 13.16	0.482
T-Cho (mg/dL)	191.50 ± 6.30	168.64 ± 6.28	0.020 *
HbA1c (NGSP) (%)	5.33 ± 0.09	5.13 ± 0.04	0.048 *
AST (GOT) (U/L)	23.31 ± 4.89	16.73 ± 1.60	0.289
ALT (GPT) (U/L)	36.81 ± 17.44	16.91 ± 3.93	0.361
γ-GTP (U/L)	29.06 ± 7.40	22.64 ± 4.24	0.511
WBC (μL)	6137.50 ± 337.25.64	6000.00 ± 463.39	0.808
RBC (×10^4^/μL)	467.69 ± 12.55	479.45 ± 11.90	0.522
Hb (g/dL)	13.83 ± 0.46	14.28 ± 0.34	0.471
HT (%)	41.76 ± 1.14	43.42 ± 0.97	0.309
MCV (fL)	89.46 ± 1.48	90.68 ± 1.24	0.560
MCH (pg)	29.59 ± 0.61	29.84 ± 0.39	0.761
MCHC (%)	33.02 ± 0.28	32.90 ± 0.18	0.752
Plt (×10^4^/μL)	26.34 ± 1.30	29.40 ± 1.29	0.118
hs-CRP (ng/mL)	1197.38 ± 436.07	134.44 ± 40.53	0.034 *

Data are shown in mean ± standard error. *t*-test. HDL, High-Density Lipoprotein; LDL, Low-Density Lipoprotein; LDL/HDL ratio, Low-Density Lipoprotein/High-Density Lipoprotein ratio; MDA-LDL, Malondialdehyde-modified LDL; TG, Triglyceride; T-Chol, Total Cholesterol; HbA1c (NGSP), Hemoglobin A1c (National Glycohemoglobin Standardization Program); AST (GOT), Aspartate transaminase (Glutamic Oxaloacetic Transaminase); ALT (GPT), Alanine transaminase (Glutamic Pyruvic Transaminase); γ-GTP, γ-glutamyl transpeptidase; WBC, Leukocyte; RBC, Erythrocyte; Hb, Hemoglobin; HT, Hematocrit; MCV, mean corpuscular volume; MCH, mean corpuscular hemoglobin; MCHC, mean corpuscular hemoglobin concentration; Plt, Plate; hs-CRP, high sensitivity C-reactive protein. * *p* < 0.05.

**Table 5:** Correlation between hs-CRP and the other systemic data.

Factors	*r*	*p*
HDL (mg/dL)	−0.389	0.045 *
LDL (mg/dL)	0.625	<0.001 **
LDL/HDL ratio	0.748	<0.001 **
MDA-LDL (U/L)	0.468	0.014 *
TG (mg/dL)	0.243	0.222
T-Cho (mg/dL)	0.511	0.006 **
HbA1c (NGSP) (%)	0.374	0.054
AST(GOT) (U/L)	0.376	0.053
ALT(GPT) (U/L)	0.412	0.033 *
γ-GTP (U/L)	0.585	0.001 **

Pearson product-moment correlation coefficient. hs-CRP, high sensitivity C-reactive protein; HDL, High-Density Lipoprotein; LDL, Low-Density Lipoprotein; LDL/HDL ratio, Low-Density Lipoprotein/High-Density Lipoprotein ratio; MDA-LDL, Malondialdehyde-modified LDL; TG, Triglyceride; T-Chol, Total Cholesterol; HbA1c (NGSP), Hemoglobin A1c (National Glycohemoglobin Standardization Program); AST (GOT), Aspartate transaminase (Glutamic Oxaloacetic Transaminase); ALT (GPT), Alanine transaminase (Glutamic Pyruvic Transaminase); γ-GTP, γ-glutamyl transpeptidase. * *p* < 0.05, ** *p* < 0.01.

**Table 6:** Ingestion Frequency Investigation with or without antioxidative supplement intake.

Factors	Non-Supplement Intake Group (*n* = 16)	Supplement Intake Group (*n* = 11)	*p*
Energy (kcal/day)	1712.40 ± 183.28	1635.25 ± 254.15	0.802
Protein (g/day)	60.45 ± 6.49	51.58 ± 10.41	0.453
Total fat (g/day)	58.25 ± 6.84	51.33 ± 12.35	0.602
Carbohydrate (g/day)	221.65 ± 27.20	192.53 ± 27.87	0.475
Retinol (mg/day)	382.07 ± 126.67	459.74 ± 170.71	0.712
Retinol Equivalent (μgRAE)	553.47 ± 135.05	548.98 ± 175.03	0.984
Vitamin D (μg/day)	6.64 ± 1.19	3.37 ± 0.73	0.047 *
SFA (g/day)	18.43 ± 2.41	15.71 ± 3.91	0.537
MUFA (g/day)	21.82 ± 2.41	20.48 ± 5.28	0.801
PUFA (g/day)	11.21 ± 1.27	9.54 ± 1.98	0.463
Cholesterol (mg/day)	373.72 ± 86.81	233.87 ± 52.59	0.231
n-3PUFA (g/day)	1.89 ± 0.28	1.52 ± 0.35	0.407
n-6 PUFA (g/day)	9.28 ± 1.00	7.99 ± 1.63	0.482
Lycopene (mg/day)	2959.97 ± 1016.85	2511.61 ± 1064.93	0.769
α-carotene (μg/day)	264.76 ± 74.70	122.18 ± 37.19	0.149
β-carotene (μg/day)	1422.06 ± 318.29	891.53 ± 176.60	0.210
α-tocopherol (mg/day)	5.90 ± 0.74	4.68 ± 0.88	0.302
β-tocopherol (mg/day)	0.32 ± 0.03	0.27 ± 0.06	0.490
γ-tocopherol (mg/day)	8.25 ± 1.15	7.47 ± 1.92	0.713
δ-tocopherol (mg/day)	1.82 ± 0.27	1.55 ± 0.39	0.562

*t*-test. SFA, Saturated fatty acid; PUFA, polyunsaturated fatty acid; MUFA, Monounsaturated fatty acid. * *p* < 0.05.

## Abbreviations

ALT (GPT): Alanine transaminase (Glutamic Pyruvic Transaminase); AST (GOT), Aspartate transaminase (Glutamic Oxaloacetic Transaminase); AMD, age-related macular degeneration; BCVA, best-corrected visual acuity; CVD, cardiovascular disease; FVA, functional visual acuity; γ-GTP, γ-glutamyl transpeptidase; HDL, High Density Lipoprotein; HbA1c (NGSP), Hemoglobin A1c (National Glycohemoglobin Standardization Program); high-sensitivity C-reactive protein (hs-CRP); Hb, Hemoglobin; HT, Hematocrit; IOP, intraocular pressure; IRL, intraretinal layer; LDL, Low Density Lipoprotein; LDL/ HDL ratio, Low Density Lipoprotein/ High Density Lipoprotein ratio; MCH, mean corpuscular hemoglobin; MCHC, mean corpuscular hemoglobin concentration; MCV, mean corpuscular volume; MDA-LDL, Malondialdehyde-modified LDL; MV, macular volume; OCT, Optical coherence tomography; ORL, Outer retinal layer; Plt, Plate; RBC, Erythrocyte; RPE, retinal pigment epithelium; SE, standard error; VA, visual acuity; VMR, visual maintenance ratio; T-Chol, Total Cholesterol; TG, Triglyceride; WBC, Leukocyte.
